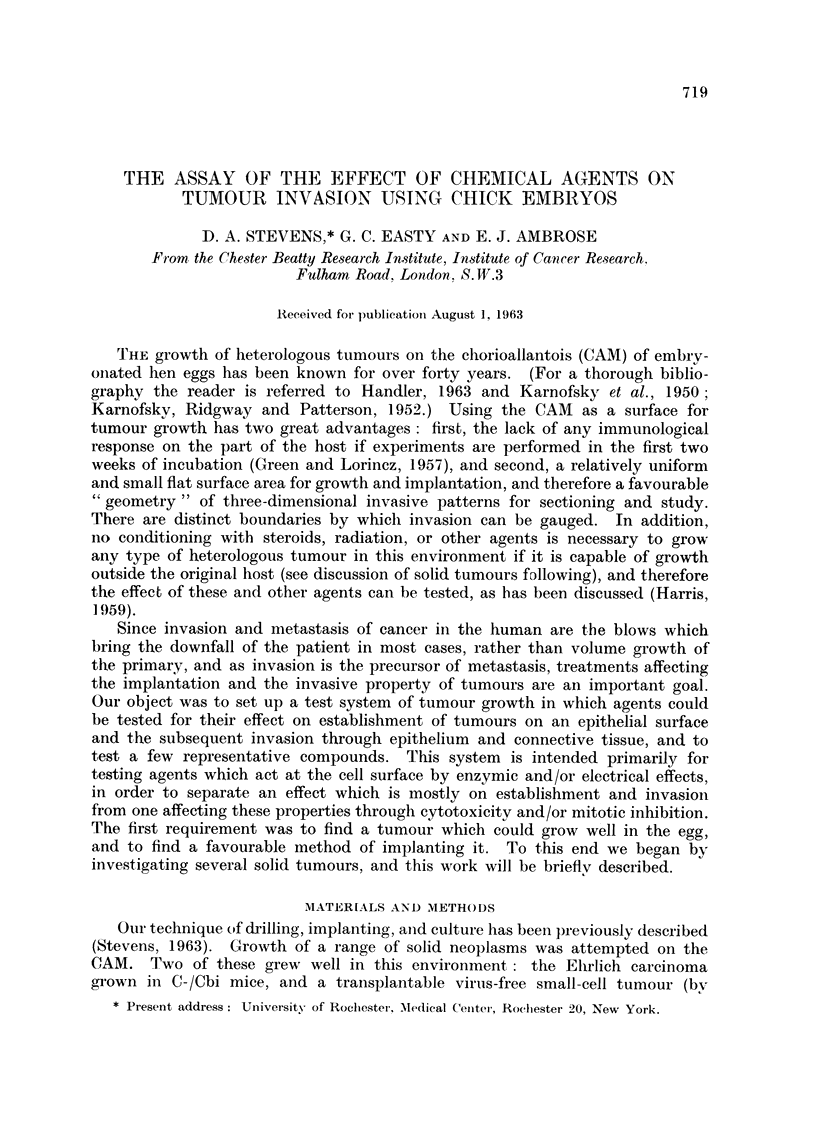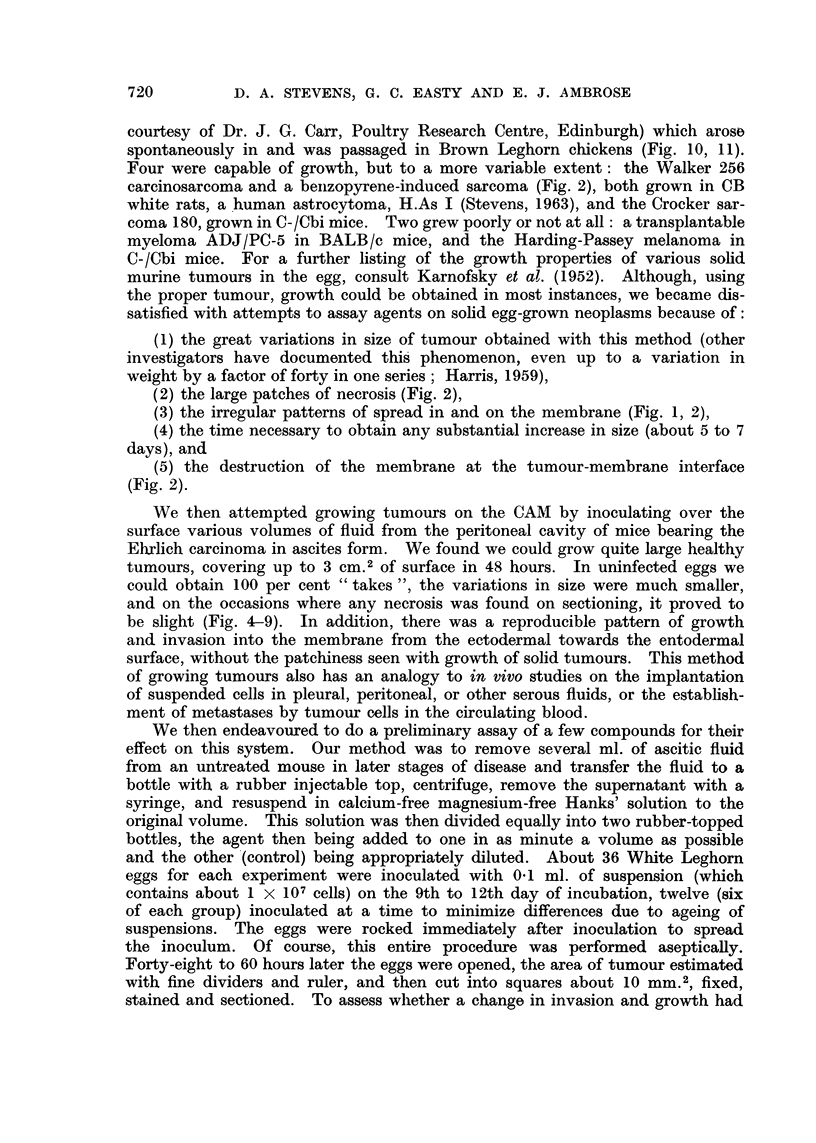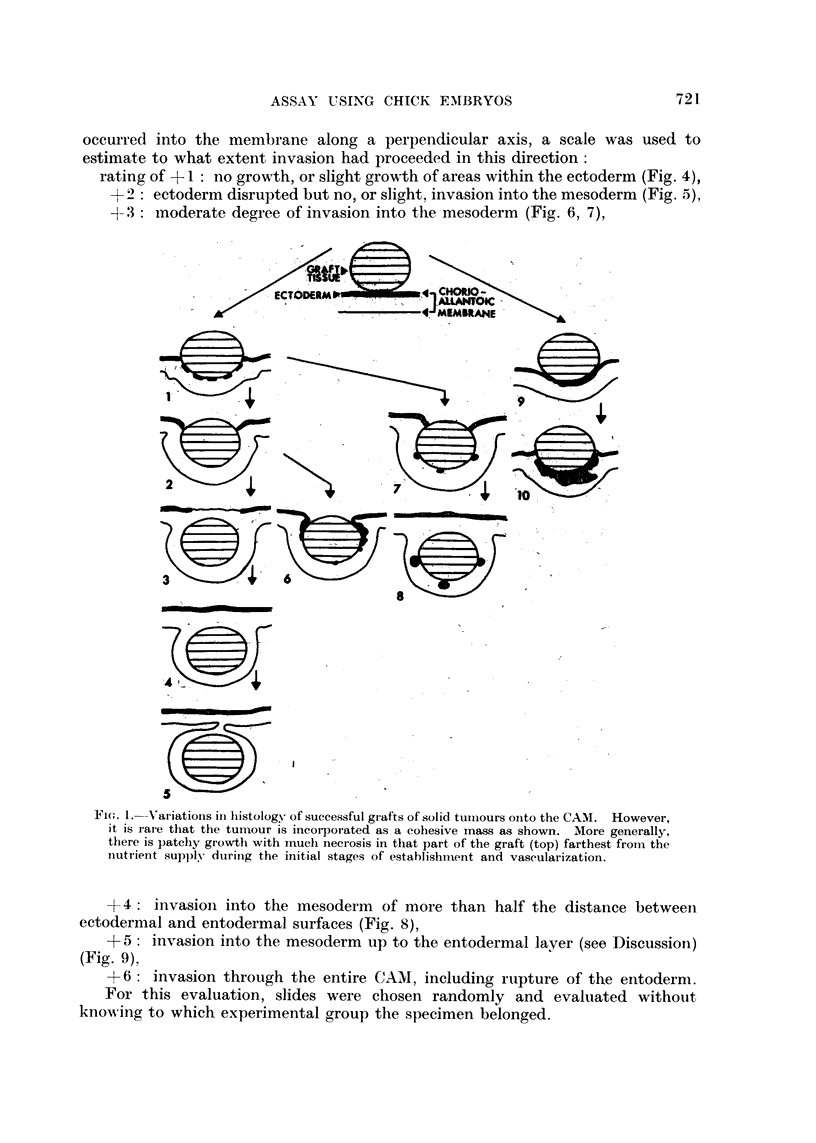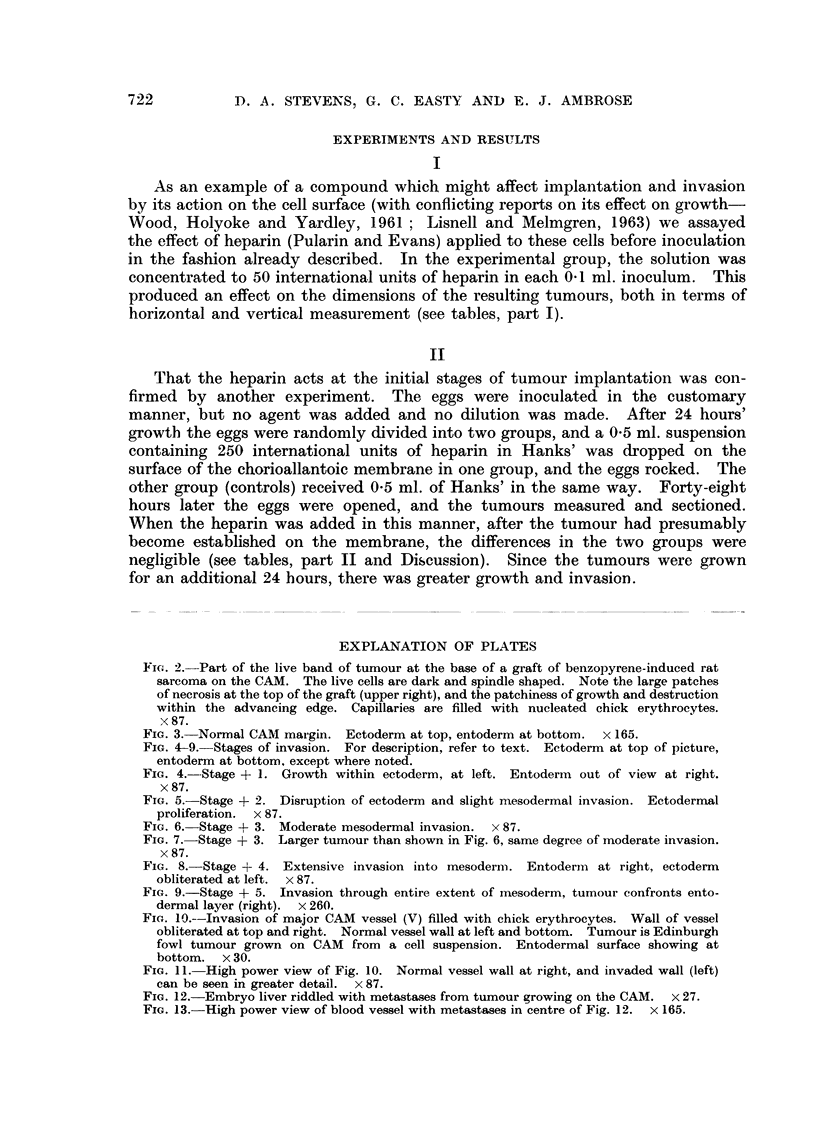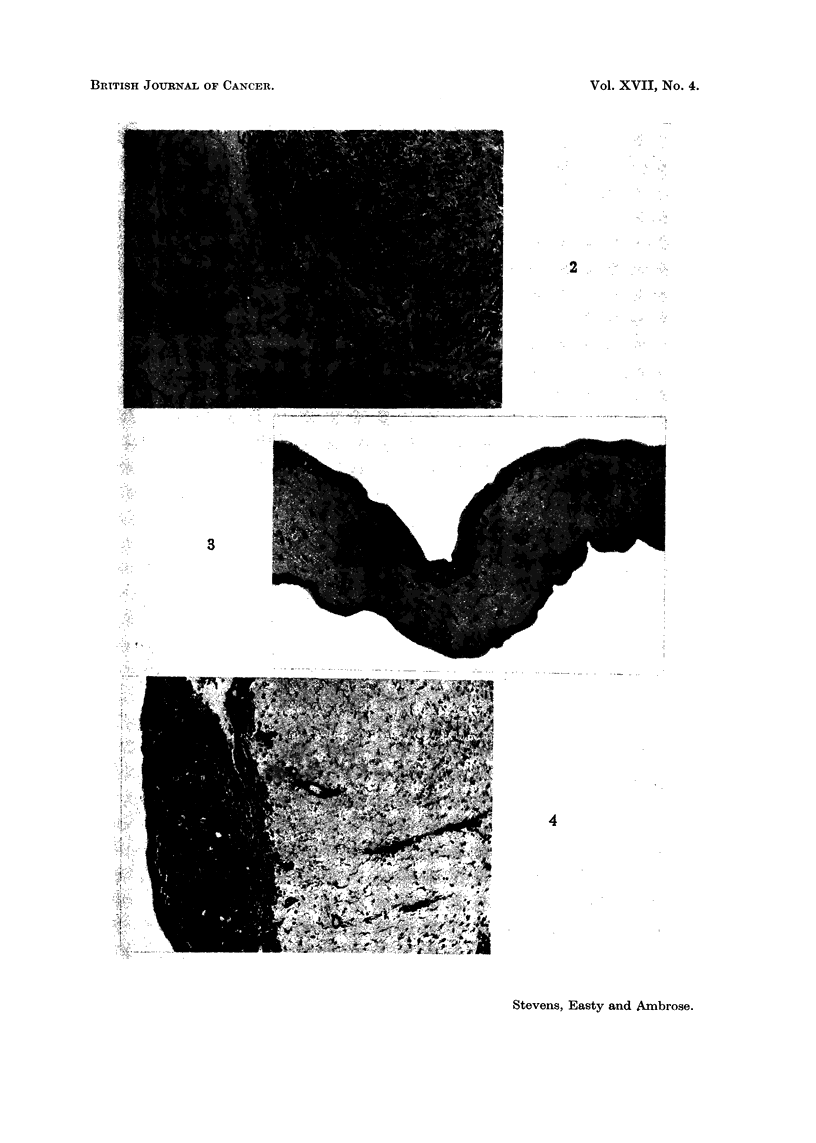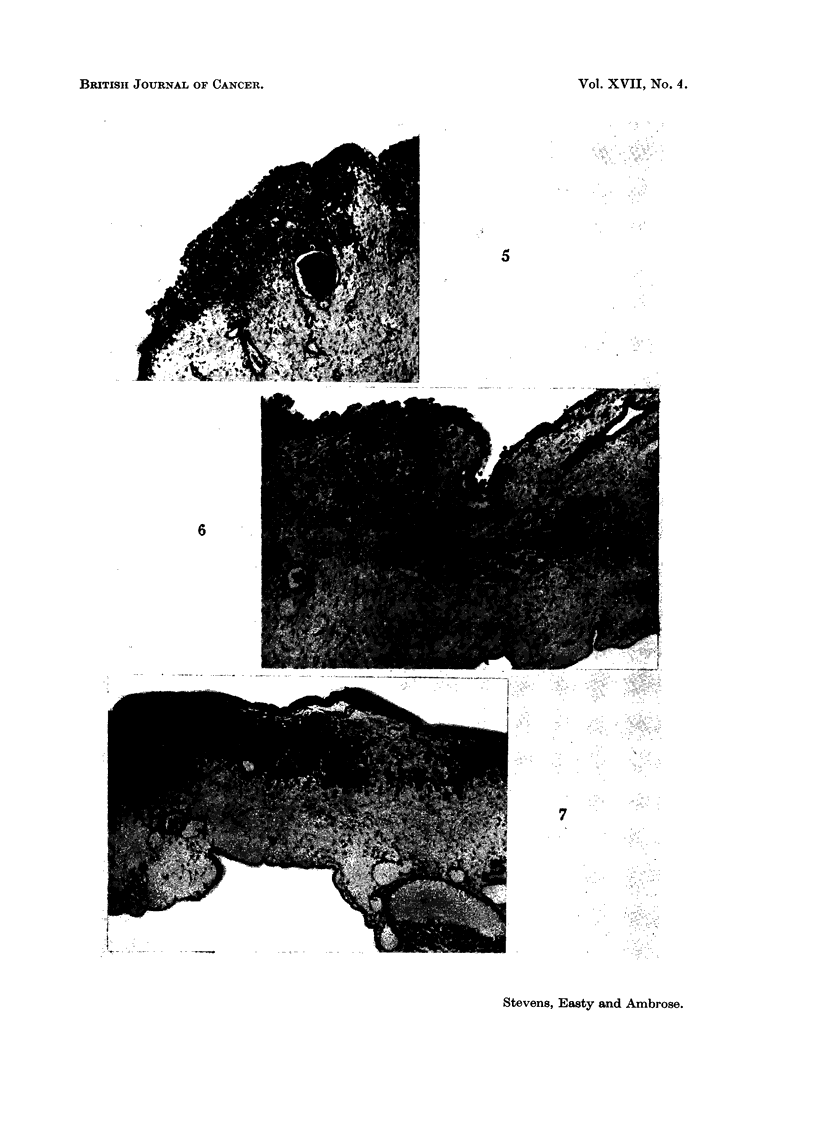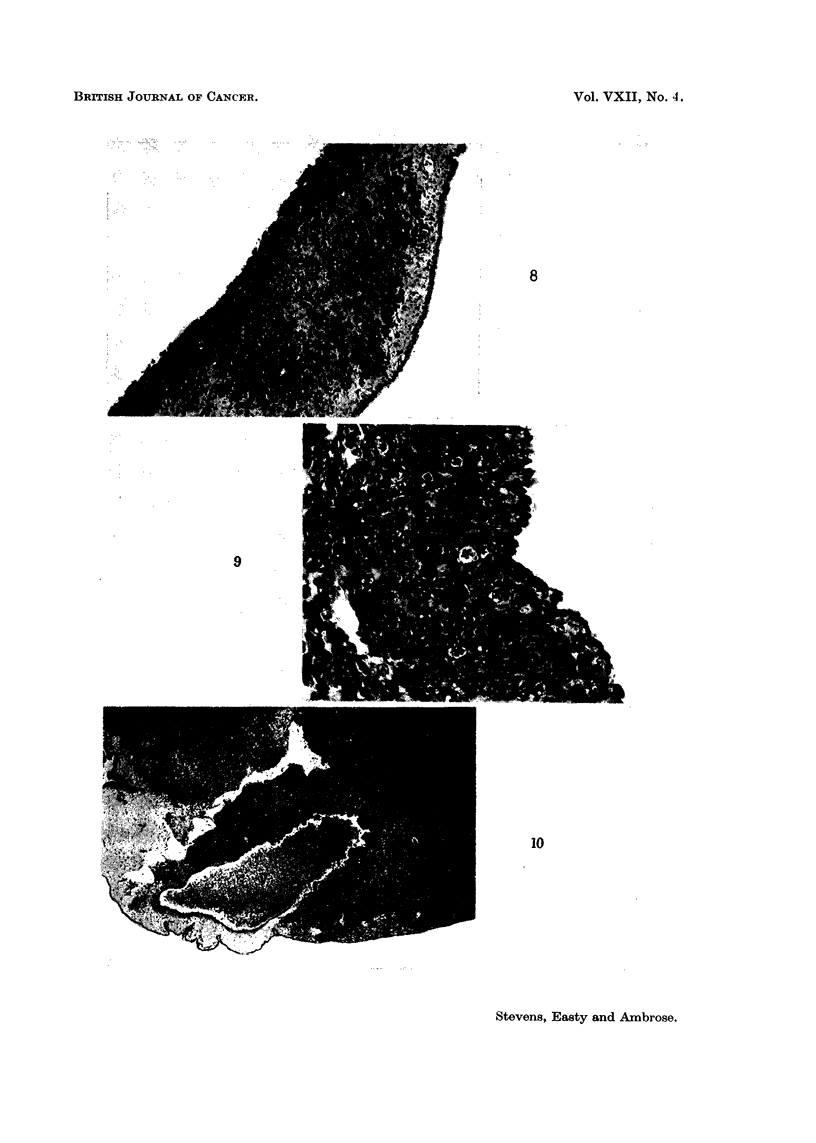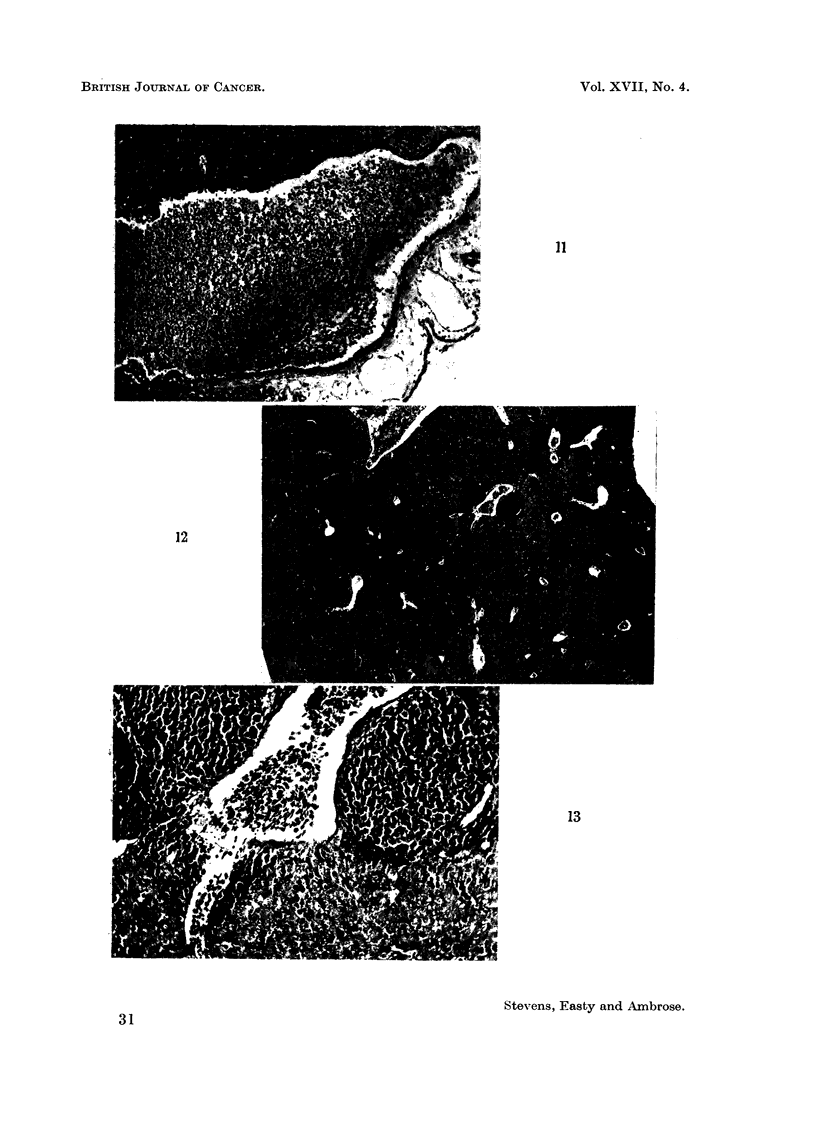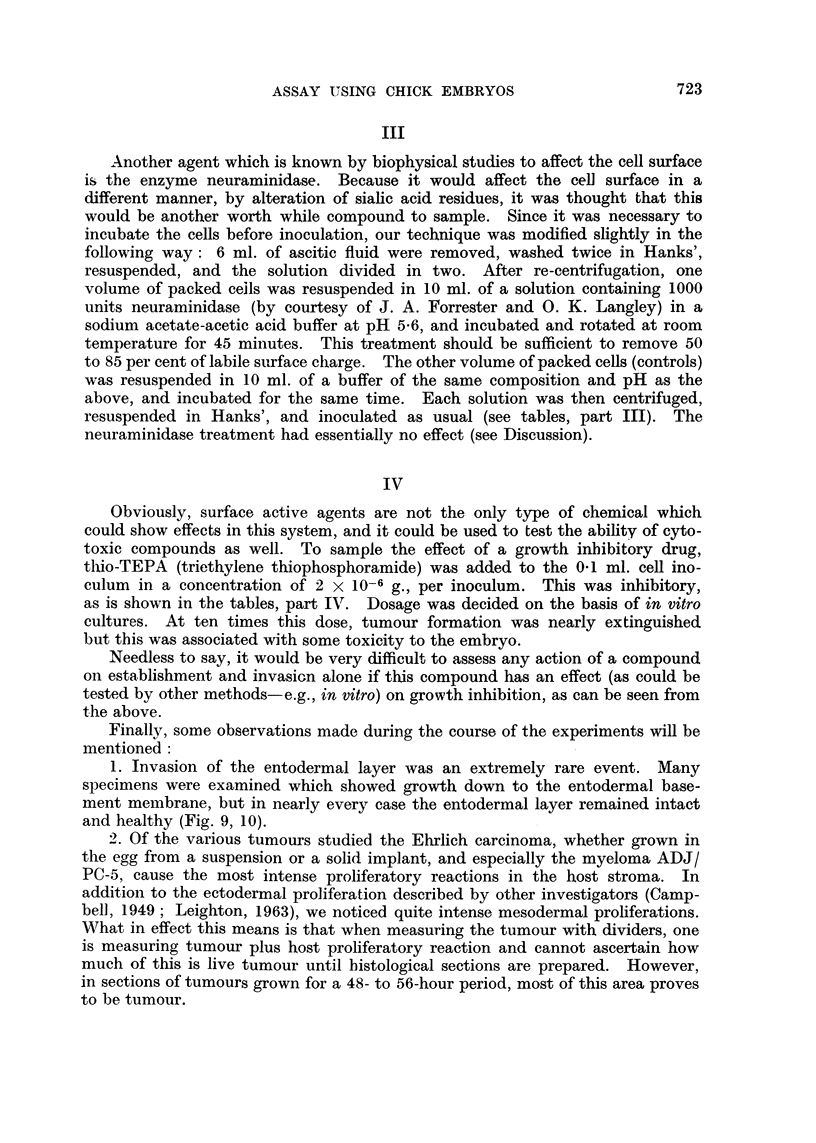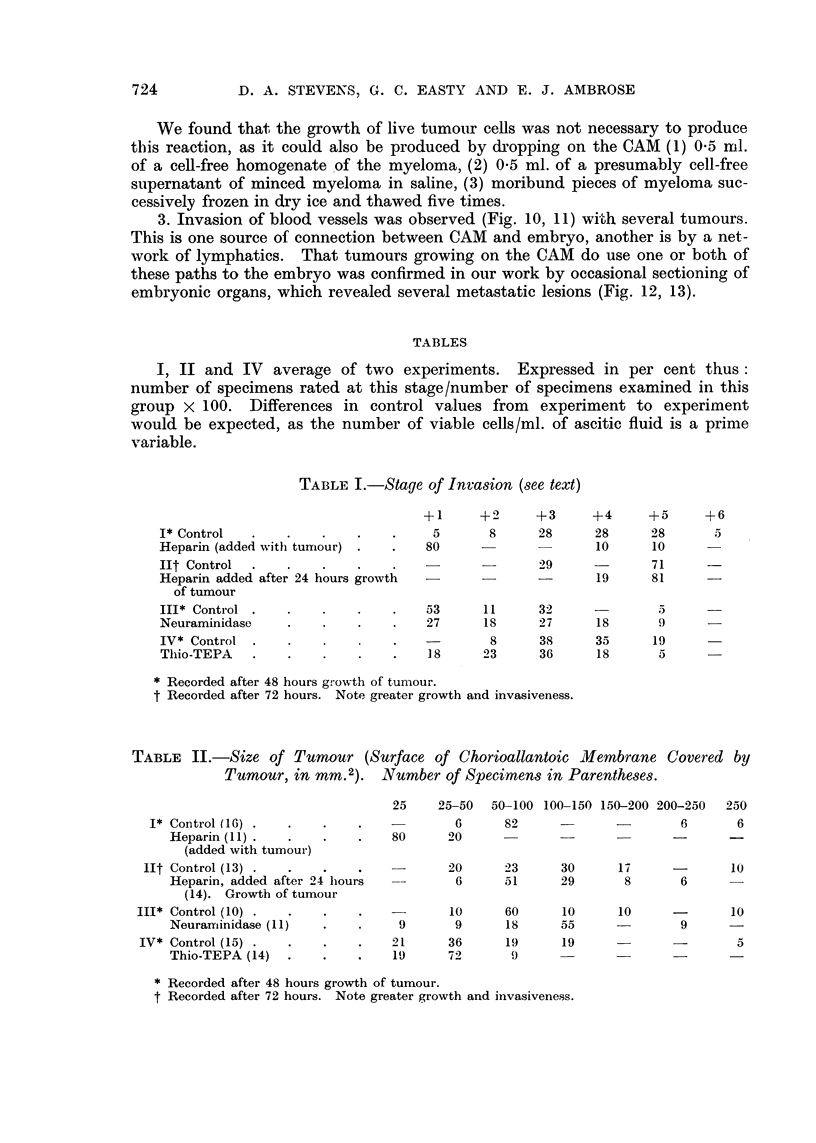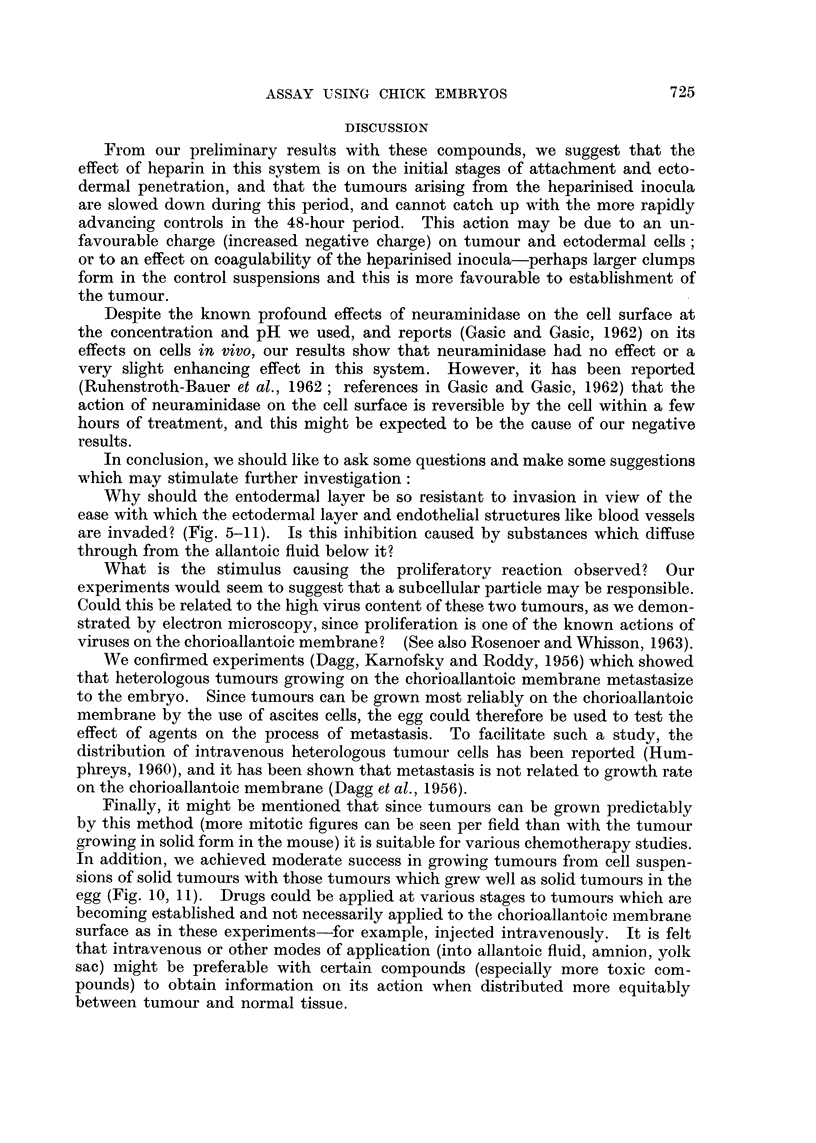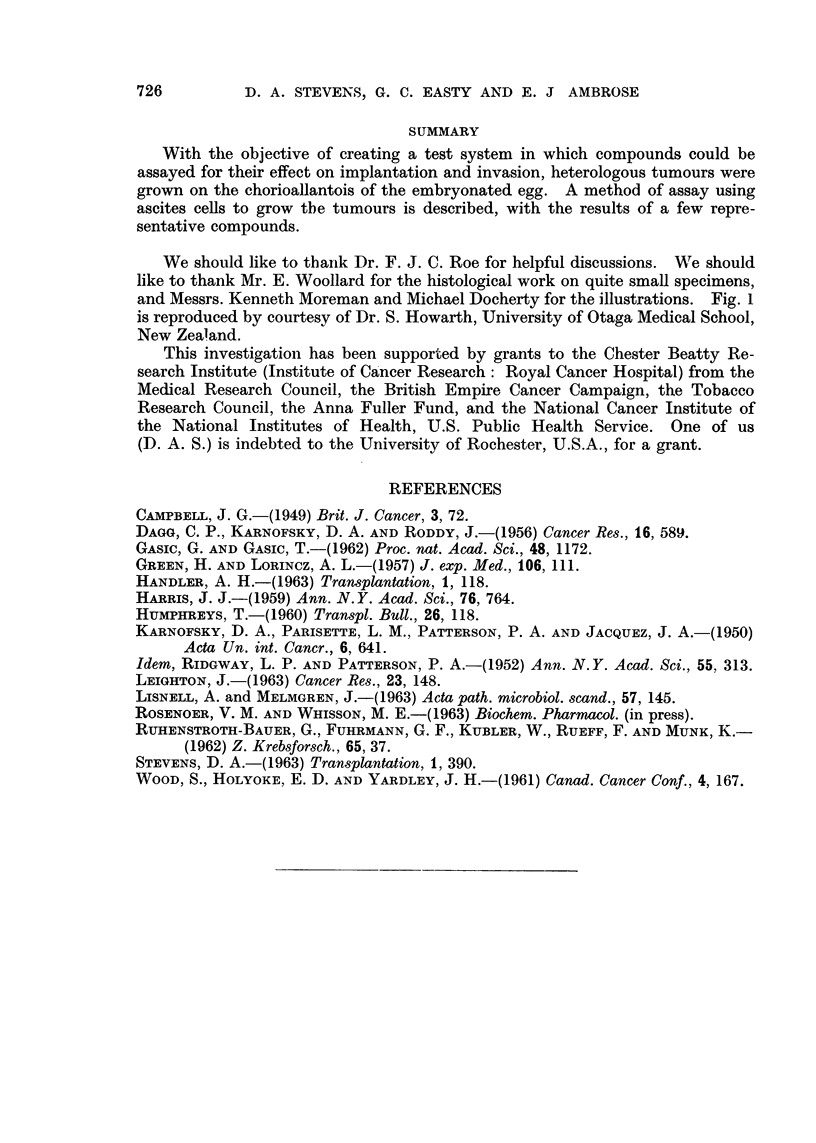# The Assay of the Effect of Chemical Agents on Tumour Invasion Using Chick Embryos

**DOI:** 10.1038/bjc.1963.92

**Published:** 1963-12

**Authors:** D. A. Stevens, G. C. Easty, E. J. Ambrose

## Abstract

**Images:**


### THE ASSAY OF THE EFFECT OF CHEMICAL AGENTS ON TUMOUR INVASION USING CHICK EMBRYOS

D. A. STEVENS,* G. C. EASTY AND E. J. AMBROSE

*From the Chester Beatty Research Institute, Institute of Cancer Research, Fulham Road, London, S.W.3*

Received for publication August 1, 1963

THE growth of heterologous tumours on the chorioallantois (CAM) of embryonated hen eggs has been known for over forty years. (For a thorough bibliography the reader is referred to Handler, 1963 and Karnofsky *et al.*, 1950; Karnofsky, Ridgway and Patterson, 1952.) Using the CAM as a surface for tumour growth has two great advantages: first, the lack of any immunological response on the part of the host if experiments are performed in the first two weeks of incubation (Green and Lorincz, 1957), and second, a relatively uniform and small flat surface area for growth and implantation, and therefore a favourable "geometry" of three-dimensional invasive patterns for sectioning and study. There are distinct boundaries by which invasion can be gauged. In addition, no conditioning with steroids, radiation, or other agents is necessary to grow any type of heterologous tumour in this environment if it is capable of growth outside the original host (see discussion of solid tumours following), and therefore the effect of these and other agents can be tested, as has been discussed (Harris, 1959).

Since invasion and metastasis of cancer in the human are the blows which bring the downfall of the patient in most cases, rather than volume growth of the primary, and as invasion is the precursor of metastasis, treatments affecting the implantation and the invasive property of tumours are an important goal. Our object was to set up a test system of tumour growth in which agents could be tested for their effect on establishment of tumours on an epithelial surface and the subsequent invasion through epithelium and connective tissue, and to test a few representative compounds. This system is intended primarily for testing agents which act at the cell surface by enzymic and/or electrical effects, in order to separate an effect which is mostly on establishment and invasion from one affecting these properties through cytotoxicity and/or mitotic inhibition. The first requirement was to find a tumour which could grow well in the egg, and to find a favourable method of implanting it. To this end we began by investigating several solid tumours, and this work will be briefly described.

### MATERIALS AND METHODS

Our technique of drilling, implanting, and culture has been previously described (Stevens, 1963). Growth of a range of solid neoplasms was attempted on the CAM. Two of these grew well in this environment: the Ehrlich carcinoma grown in C-/Cbi mice, and a transplantable virus-free small-cell tumour (by

* Present address: University of Rochester, Medical Center, Rochester 20, New York.

courtesy of Dr. J. G. Carr, Poultry Research Centre, Edinburgh) which arose spontaneously in and was passaged in Brown Leghorn chickens (Fig. 10, 11). Four were capable of growth, but to a more variable extent: the Walker 256 carcinosarcoma and a benzopyrene-induced sarcoma (Fig. 2), both grown in CB white rats, a human astrocytoma, H.As I (Stevens, 1963), and the Crocker sarcoma 180, grown in C-/Cbi mice. Two grew poorly or not at all: a transplantable myeloma ADJ/PC-5 in BALB/c mice, and the Harding-Passey melanoma in C-/Cbi mice. For a further listing of the growth properties of various solid murine tumours in the egg, consult Karnofsky *et al.* (1952). Although, using the proper tumour, growth could be obtained in most instances, we became dissatisfied with attempts to assay agents on solid egg-grown neoplasms because of:

(1) the great variations in size of tumour obtained with this method (other investigators have documented this phenomenon, even up to a variation in weight by a factor of forty in one series; Harris, 1959),
(2) the large patches of necrosis (Fig. 2),
(3) the irregular patterns of spread in and on the membrane (Fig. 1, 2),
(4) the time necessary to obtain any substantial increase in size (about 5 to 7 days), and
(5) the destruction of the membrane at the tumour-membrane interface (Fig. 2).

We then attempted growing tumours on the CAM by inoculating over the surface various volumes of fluid from the peritoneal cavity of mice bearing the Ehrlich carcinoma in ascites form. We found we could grow quite large healthy tumours, covering up to 3 cm.$^2$ of surface in 48 hours. In uninfected eggs we could obtain 100 per cent "takes", the variations in size were much smaller, and on the occasions where any necrosis was found on sectioning, it proved to be slight (Fig. 4–9). In addition, there was a reproducible pattern of growth and invasion into the membrane from the ectodermal towards the entodermal surface, without the patchiness seen with growth of solid tumours. This method of growing tumours also has an analogy to *in vivo* studies on the implantation of suspended cells in pleural, peritoneal, or other serous fluids, or the establishment of metastases by tumour cells in the circulating blood.

We then endeavoured to do a preliminary assay of a few compounds for their effect on this system. Our method was to remove several ml. of ascitic fluid from an untreated mouse in later stages of disease and transfer the fluid to a bottle with a rubber injectable top, centrifuge, remove the supernatant with a syringe, and resuspend in calcium-free magnesium-free Hanks' solution to the original volume. This solution was then divided equally into two rubber-topped bottles, the agent then being added to one in as minute a volume as possible and the other (control) being appropriately diluted. About 36 White Leghorn eggs for each experiment were inoculated with 0·1 ml. of suspension (which contains about $1 \times 10^7$ cells) on the 9th to 12th day of incubation, twelve (six of each group) inoculated at a time to minimize differences due to ageing of suspensions. The eggs were rocked immediately after inoculation to spread the inoculum. Of course, this entire procedure was performed aseptically. Forty-eight to 60 hours later the eggs were opened, the area of tumour estimated with fine dividers and ruler, and then cut into squares about 10 mm.$^2$, fixed, stained and sectioned. To assess whether a change in invasion and growth had occurred into the membrane along a perpendicular axis, a scale was used to estimate to what extent invasion had proceeded in this direction :

rating of +1 : no growth, or slight growth of areas within the ectoderm (Fig. 4),
+2 : ectoderm disrupted but no, or slight, invasion into the mesoderm (Fig. 5),
+3 : moderate degree of invasion into the mesoderm (Fig. 6, 7),

FIG. 1.—Variations in histology of successful grafts of solid tumours onto the CAM. However, it is rare that the tumour is incorporated as a cohesive mass as shown. More generally, there is patchy growth with much necrosis in that part of the graft (top) farthest from the nutrient supply during the initial stages of establishment and vascularization.

+4 : invasion into the mesoderm of more than half the distance between ectodermal and entodermal surfaces (Fig. 8),
+5 : invasion into the mesoderm up to the entodermal layer (see Discussion) (Fig. 9),
+6 : invasion through the entire CAM, including rupture of the entoderm.

For this evaluation, slides were chosen randomly and evaluated without knowing to which experimental group the specimen belonged.

### EXPERIMENTS AND RESULTS

### I

As an example of a compound which might affect implantation and invasion by its action on the cell surface (with conflicting reports on its effect on growth—Wood, Holyoke and Yardley, 1961 ; Lisnell and Melmgren, 1963) we assayed the effect of heparin (Pularin and Evans) applied to these cells before inoculation in the fashion already described. In the experimental group, the solution was concentrated to 50 international units of heparin in each 0·1 ml. inoculum. This produced an effect on the dimensions of the resulting tumours, both in terms of horizontal and vertical measurement (see tables, part I).

### II

That the heparin acts at the initial stages of tumour implantation was confirmed by another experiment. The eggs were inoculated in the customary manner, but no agent was added and no dilution was made. After 24 hours' growth the eggs were randomly divided into two groups, and a 0·5 ml. suspension containing 250 international units of heparin in Hanks' was dropped on the surface of the chorioallantoic membrane in one group, and the eggs rocked. The other group (controls) received 0·5 ml. of Hanks' in the same way. Forty-eight hours later the eggs were opened, and the tumours measured and sectioned. When the heparin was added in this manner, after the tumour had presumably become established on the membrane, the differences in the two groups were negligible (see tables, part II and Discussion). Since the tumours were grown for an additional 24 hours, there was greater growth and invasion.

---

### EXPLANATION OF PLATES

FIG. 2.—Part of the live band of tumour at the base of a graft of benzopyrene-induced rat sarcoma on the CAM. The live cells are dark and spindle shaped. Note the large patches of necrosis at the top of the graft (upper right), and the patchiness of growth and destruction within the advancing edge. Capillaries are filled with nucleated chick erythrocytes. ×87.

FIG. 3.—Normal CAM margin. Ectoderm at top, entoderm at bottom. ×165.

FIG. 4–9.—Stages of invasion. For description, refer to text. Ectoderm at top of picture, entoderm at bottom, except where noted.

FIG. 4.—Stage + 1. Growth within ectoderm, at left. Entoderm out of view at right. ×87.

FIG. 5.—Stage + 2. Disruption of ectoderm and slight mesodermal invasion. Ectodermal proliferation. ×87.

FIG. 6.—Stage + 3. Moderate mesodermal invasion. ×87.

FIG. 7.—Stage + 3. Larger tumour than shown in Fig. 6, same degree of moderate invasion. ×87.

FIG. 8.—Stage + 4. Extensive invasion into mesoderm. Entoderm at right, ectoderm obliterated at left. ×87.

FIG. 9.—Stage + 5. Invasion through entire extent of mesoderm, tumour confronts entodermal layer (right). ×260.

FIG. 10.—Invasion of major CAM vessel (V) filled with chick erythrocytes. Wall of vessel obliterated at top and right. Normal vessel wall at left and bottom. Tumour is Edinburgh fowl tumour grown on CAM from a cell suspension. Entodermal surface showing at bottom. ×30.

FIG. 11.—High power view of Fig. 10. Normal vessel wall at right, and invaded wall (left) can be seen in greater detail. ×87.

FIG. 12.—Embryo liver riddled with metastases from tumour growing on the CAM. ×27.

FIG. 13.—High power view of blood vessel with metastases in centre of Fig. 12. ×165.

2

3

4

Stevens, Easty and Ambrose.

5

6

7

Stevens, Easty and Ambrose.

8

9

10

Stevens, Easty and Ambrose.

11

12

13

Stevens, Easty and Ambrose.

### III

Another agent which is known by biophysical studies to affect the cell surface is the enzyme neuraminidase. Because it would affect the cell surface in a different manner, by alteration of sialic acid residues, it was thought that this would be another worth while compound to sample. Since it was necessary to incubate the cells before inoculation, our technique was modified slightly in the following way : 6 ml. of ascitic fluid were removed, washed twice in Hanks', resuspended, and the solution divided in two. After re-centrifugation, one volume of packed cells was resuspended in 10 ml. of a solution containing 1000 units neuraminidase (by courtesy of J. A. Forrester and O. K. Langley) in a sodium acetate-acetic acid buffer at pH 5·6, and incubated and rotated at room temperature for 45 minutes. This treatment should be sufficient to remove 50 to 85 per cent of labile surface charge. The other volume of packed cells (controls) was resuspended in 10 ml. of a buffer of the same composition and pH as the above, and incubated for the same time. Each solution was then centrifuged, resuspended in Hanks', and inoculated as usual (see tables, part III). The neuraminidase treatment had essentially no effect (see Discussion).

### IV

Obviously, surface active agents are not the only type of chemical which could show effects in this system, and it could be used to test the ability of cytotoxic compounds as well. To sample the effect of a growth inhibitory drug, thio-TEPA (triethylene thiophosphoramide) was added to the 0·1 ml. cell inoculum in a concentration of $2 \times 10^{-6}$ g., per inoculum. This was inhibitory, as is shown in the tables, part IV. Dosage was decided on the basis of *in vitro* cultures. At ten times this dose, tumour formation was nearly extinguished but this was associated with some toxicity to the embryo.

Needless to say, it would be very difficult to assess any action of a compound on establishment and invasion alone if this compound has an effect (as could be tested by other methods—e.g., *in vitro*) on growth inhibition, as can be seen from the above.

Finally, some observations made during the course of the experiments will be mentioned :

1. Invasion of the entodermal layer was an extremely rare event. Many specimens were examined which showed growth down to the entodermal basement membrane, but in nearly every case the entodermal layer remained intact and healthy (Fig. 9, 10).

2. Of the various tumours studied the Ehrlich carcinoma, whether grown in the egg from a suspension or a solid implant, and especially the myeloma ADJ/PC-5, cause the most intense proliferatory reactions in the host stroma. In addition to the ectodermal proliferation described by other investigators (Campbell, 1949 ; Leighton, 1963), we noticed quite intense mesodermal proliferations. What in effect this means is that when measuring the tumour with dividers, one is measuring tumour plus host proliferatory reaction and cannot ascertain how much of this is live tumour until histological sections are prepared. However, in sections of tumours grown for a 48- to 56-hour period, most of this area proves to be tumour.

We found that the growth of live tumour cells was not necessary to produce this reaction, as it could also be produced by dropping on the CAM (1) 0·5 ml. of a cell-free homogenate of the myeloma, (2) 0·5 ml. of a presumably cell-free supernatant of minced myeloma in saline, (3) moribund pieces of myeloma successively frozen in dry ice and thawed five times.

3. Invasion of blood vessels was observed (Fig. 10, 11) with several tumours. This is one source of connection between CAM and embryo, another is by a network of lymphatics. That tumours growing on the CAM do use one or both of these paths to the embryo was confirmed in our work by occasional sectioning of embryonic organs, which revealed several metastatic lesions (Fig. 12, 13).

### TABLES

I, II and IV average of two experiments. Expressed in per cent thus: number of specimens rated at this stage/number of specimens examined in this group × 100. Differences in control values from experiment to experiment would be expected, as the number of viable cells/ml. of ascitic fluid is a prime variable.

TABLE I.—*Stage of Invasion* (*see text*)

| | +1 | +2 | +3 | +4 | +5 | +6 |
|---|---|---|---|---|---|---|
| I* Control . . . . . . | 5 | 8 | 28 | 28 | 28 | 5 |
| Heparin (added with tumour) . . | 80 | — | — | 10 | 10 | — |
| II† Control . . . . . . | — | — | 29 | — | 71 | — |
| Heparin added after 24 hours growth of tumour | — | — | — | 19 | 81 | — |
| III* Control . . . . . . | 53 | 11 | 32 | — | 5 | — |
| Neuraminidase . . . . | 27 | 18 | 27 | 18 | 9 | — |
| IV* Control . . . . . . | — | 8 | 38 | 35 | 19 | — |
| Thio-TEPA . . . . . . | 18 | 23 | 36 | 18 | 5 | — |

* Recorded after 48 hours growth of tumour.
† Recorded after 72 hours. Note greater growth and invasiveness.

TABLE II.—*Size of Tumour* (*Surface of Chorioallantoic Membrane Covered by Tumour, in mm.*$^2$). *Number of Specimens in Parentheses.*

| | | 25 | 25–50 | 50–100 | 100–150 | 150–200 | 200–250 | 250 |
|---|---|---|---|---|---|---|---|---|
| I* | Control (16) . . . . | — | 6 | 82 | — | — | 6 | 6 |
| | Heparin (11) . . . . (added with tumour) | 80 | 20 | — | — | — | — | — |
| II† | Control (13) . . . . | — | 20 | 23 | 30 | 17 | — | 10 |
| | Heparin, added after 24 hours (14). Growth of tumour | — | 6 | 51 | 29 | 8 | 6 | — |
| III* | Control (10) . . . . | — | 10 | 60 | 10 | 10 | — | 10 |
| | Neuraminidase (11) . . | 9 | 9 | 18 | 55 | — | 9 | — |
| IV* | Control (15) . . . . | 21 | 36 | 19 | 19 | — | — | 5 |
| | Thio-TEPA (14) . . . | 19 | 72 | 9 | — | — | — | — |

* Recorded after 48 hours growth of tumour.
† Recorded after 72 hours. Note greater growth and invasiveness.

### DISCUSSION

From our preliminary results with these compounds, we suggest that the effect of heparin in this system is on the initial stages of attachment and ectodermal penetration, and that the tumours arising from the heparinised inocula are slowed down during this period, and cannot catch up with the more rapidly advancing controls in the 48-hour period. This action may be due to an unfavourable charge (increased negative charge) on tumour and ectodermal cells ; or to an effect on coagulability of the heparinised inocula—perhaps larger clumps form in the control suspensions and this is more favourable to establishment of the tumour.

Despite the known profound effects of neuraminidase on the cell surface at the concentration and pH we used, and reports (Gasic and Gasic, 1962) on its effects on cells *in vivo*, our results show that neuraminidase had no effect or a very slight enhancing effect in this system. However, it has been reported (Ruhenstroth-Bauer *et al.*, 1962 ; references in Gasic and Gasic, 1962) that the action of neuraminidase on the cell surface is reversible by the cell within a few hours of treatment, and this might be expected to be the cause of our negative results.

In conclusion, we should like to ask some questions and make some suggestions which may stimulate further investigation :

Why should the entodermal layer be so resistant to invasion in view of the ease with which the ectodermal layer and endothelial structures like blood vessels are invaded? (Fig. 5–11). Is this inhibition caused by substances which diffuse through from the allantoic fluid below it?

What is the stimulus causing the proliferatory reaction observed? Our experiments would seem to suggest that a subcellular particle may be responsible. Could this be related to the high virus content of these two tumours, as we demonstrated by electron microscopy, since proliferation is one of the known actions of viruses on the chorioallantoic membrane? (See also Rosenoer and Whisson, 1963).

We confirmed experiments (Dagg, Karnofsky and Roddy, 1956) which showed that heterologous tumours growing on the chorioallantoic membrane metastasize to the embryo. Since tumours can be grown most reliably on the chorioallantoic membrane by the use of ascites cells, the egg could therefore be used to test the effect of agents on the process of metastasis. To facilitate such a study, the distribution of intravenous heterologous tumour cells has been reported (Humphreys, 1960), and it has been shown that metastasis is not related to growth rate on the chorioallantoic membrane (Dagg *et al.*, 1956).

Finally, it might be mentioned that since tumours can be grown predictably by this method (more mitotic figures can be seen per field than with the tumour growing in solid form in the mouse) it is suitable for various chemotherapy studies. In addition, we achieved moderate success in growing tumours from cell suspensions of solid tumours with those tumours which grew well as solid tumours in the egg (Fig. 10, 11). Drugs could be applied at various stages to tumours which are becoming established and not necessarily applied to the chorioallantoic membrane surface as in these experiments—for example, injected intravenously. It is felt that intravenous or other modes of application (into allantoic fluid, amnion, yolk sac) might be preferable with certain compounds (especially more toxic compounds) to obtain information on its action when distributed more equitably between tumour and normal tissue.

SUMMARY

With the objective of creating a test system in which compounds could be assayed for their effect on implantation and invasion, heterologous tumours were grown on the chorioallantois of the embryonated egg. A method of assay using ascites cells to grow the tumours is described, with the results of a few representative compounds.

We should like to thank Dr. F. J. C. Roe for helpful discussions. We should like to thank Mr. E. Woollard for the histological work on quite small specimens, and Messrs. Kenneth Moreman and Michael Docherty for the illustrations. Fig. 1 is reproduced by courtesy of Dr. S. Howarth, University of Otaga Medical School, New Zealand.

This investigation has been supported by grants to the Chester Beatty Research Institute (Institute of Cancer Research : Royal Cancer Hospital) from the Medical Research Council, the British Empire Cancer Campaign, the Tobacco Research Council, the Anna Fuller Fund, and the National Cancer Institute of the National Institutes of Health, U.S. Public Health Service. One of us (D. A. S.) is indebted to the University of Rochester, U.S.A., for a grant.

### REFERENCES

CAMPBELL, J. G.—(1949) *Brit. J. Cancer*, **3**, 72.
DAGG, C. P., KARNOFSKY, D. A. AND RODDY, J.—(1956) *Cancer Res.*, **16**, 589.
GASIC, G. AND GASIC, T.—(1962) *Proc. nat. Acad. Sci.*, **48**, 1172.
GREEN, H. AND LORINCZ, A. L.—(1957) *J. exp. Med.*, **106**, 111.
HANDLER, A. H.—(1963) *Transplantation*, **1**, 118.
HARRIS, J. J.—(1959) *Ann. N.Y. Acad. Sci.*, **76**, 764.
HUMPHREYS, T.—(1960) *Transpl. Bull.*, **26**, 118.
KARNOFSKY, D. A., PARISETTE, L. M., PATTERSON, P. A. AND JACQUEZ, J. A.—(1950) *Acta Un. int. Cancr.*, **6**, 641.
*Idem*, RIDGWAY, L. P. AND PATTERSON, P. A.—(1952) *Ann. N.Y. Acad. Sci.*, **55**, 313.
LEIGHTON, J.—(1963) *Cancer Res.*, **23**, 148.
LISNELL, A. and MELMGREN, J.—(1963) *Acta path. microbiol. scand.*, **57**, 145.
ROSENOER, V. M. AND WHISSON, M. E.—(1963) *Biochem. Pharmacol.* (in press).
RUHENSTROTH-BAUER, G., FUHRMANN, G. F., KUBLER, W., RUEFF, F. AND MUNK, K.—(1962) *Z. Krebsforsch.*, **65**, 37.
STEVENS, D. A.—(1963) *Transplantation*, **1**, 390.
WOOD, S., HOLYOKE, E. D. AND YARDLEY, J. H.—(1961) *Canad. Cancer Conf.*, **4**, 167.